# Loop-and-let-go technique for the management of symptomatic large colonic lipoma

**DOI:** 10.1016/j.vgie.2023.04.003

**Published:** 2023-06-08

**Authors:** Syedreza Haider, Matthew Peller, Vladimir Lamm, Ahmad Najdat Bazarbashi

**Affiliations:** Division of Gastroenterology, Washington University School of Medicine, St. Louis, Missouri

## Abstract

Video 1 We depict (1) identification of a large lipoma in the sigmoid colon; (2) radial echo endoscopy confirming diagnosis; (3) removal with the loop-and-let-go technique; (4) ex vivo demonstration of the technique; and (5) an interval follow-up demonstrating healing of the lesion.

We depict (1) identification of a large lipoma in the sigmoid colon; (2) radial echo endoscopy confirming diagnosis; (3) removal with the loop-and-let-go technique; (4) ex vivo demonstration of the technique; and (5) an interval follow-up demonstrating healing of the lesion.

## Introduction

Colonic lipomas are adipose tumors that originate from the submucosal layer. While often asymptomatic, larger lipomas can cause symptoms such as abdominal pain and changes in bowel habits, thought to be secondary to intermittent intussusception.^1^^,^^2^ When large tumors begin to instigate obstructive symptoms, resection is warranted to alleviate symptom burden.^1^^,^^3^ While smaller lipomas can be removed endoscopically, larger ones often carry risk of adverse events from electrocautery such as hot snare polypectomy or EMR.^1^^,^^4^ Recent developments in endoscopic techniques have revealed a promising alternative described as the “loop-and-let-go” technique.^1^^,^^5^ This involves looping and ligating the lipoma at the base, then subsequently allowing for auto-amputation and passage of the tumor thus ameliorating the need for electrocautery. This case describes the first video documentation of this technique removing a large colonic lipoma.

## Case Description

A 69-year-old woman presented to her gastroenterologist with several months of intermittent bloating, abdominal pain, and constipation. She denied melena, nausea, or vomiting. A thorough evaluation of her symptoms was unrevealing. The patient subsequently underwent a colonoscopy and was found to have a 50-mm lipoma identified in the sigmoid colon. Because of concern for intermittent obstruction from the large lipoma, the patient was referred for endoscopic management. The patient’s consent was obtained to perform the procedure, and risks and benefits were reviewed.

**Figure 1:**
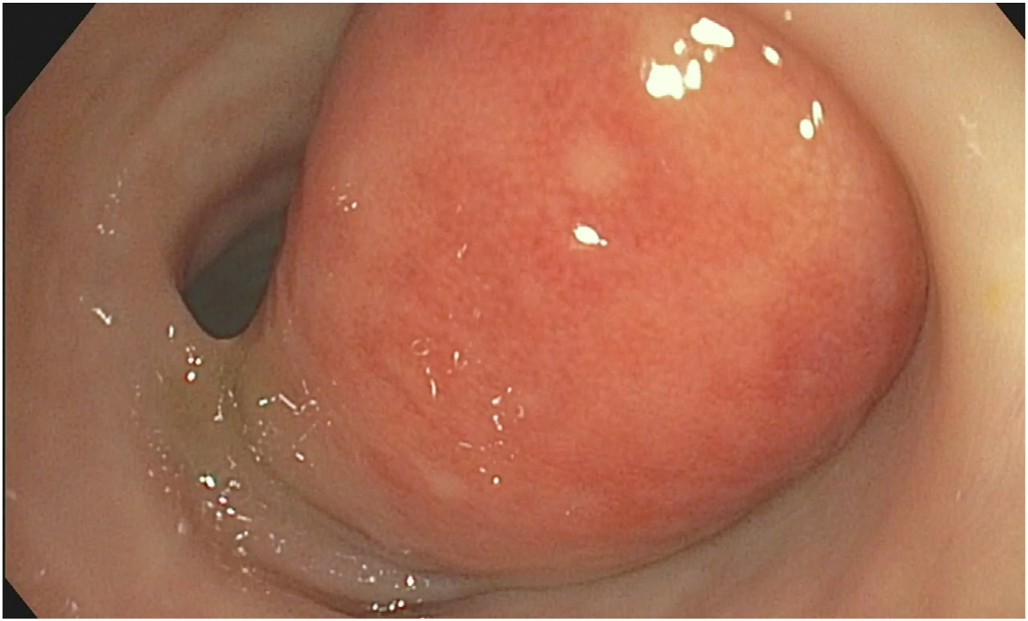
Large 50-mm sigmoid polypoid mass seen on colonoscopy.

**Figure 2:**
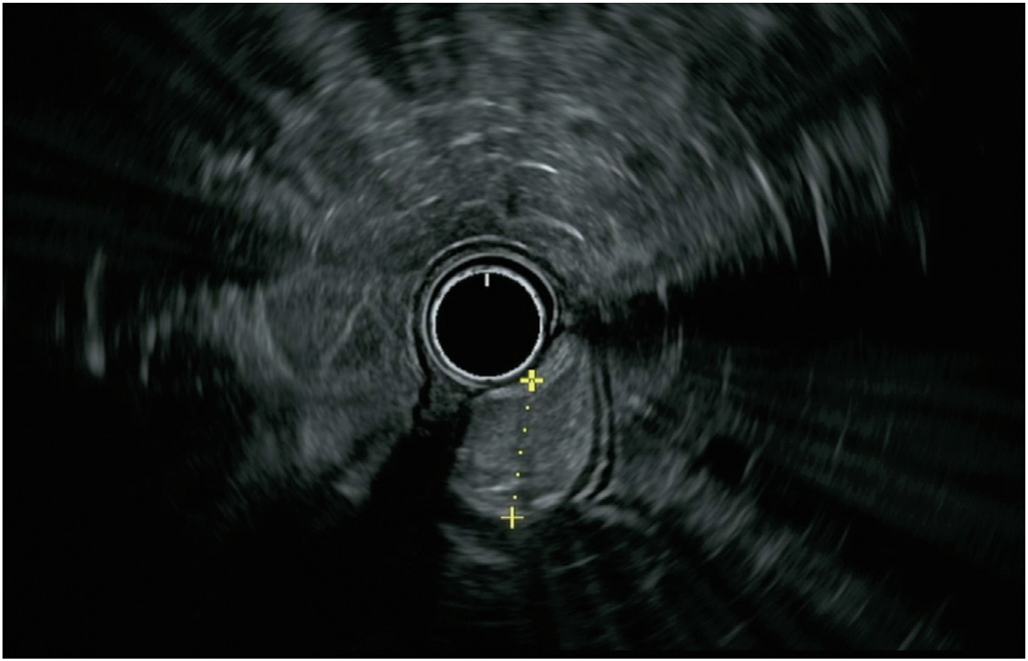
Radial EUS demonstrated a hyperechoic mass with well-defined endosonographic borders arising from the submucosal layer, consistent with lipoma.

**Figure 3:**
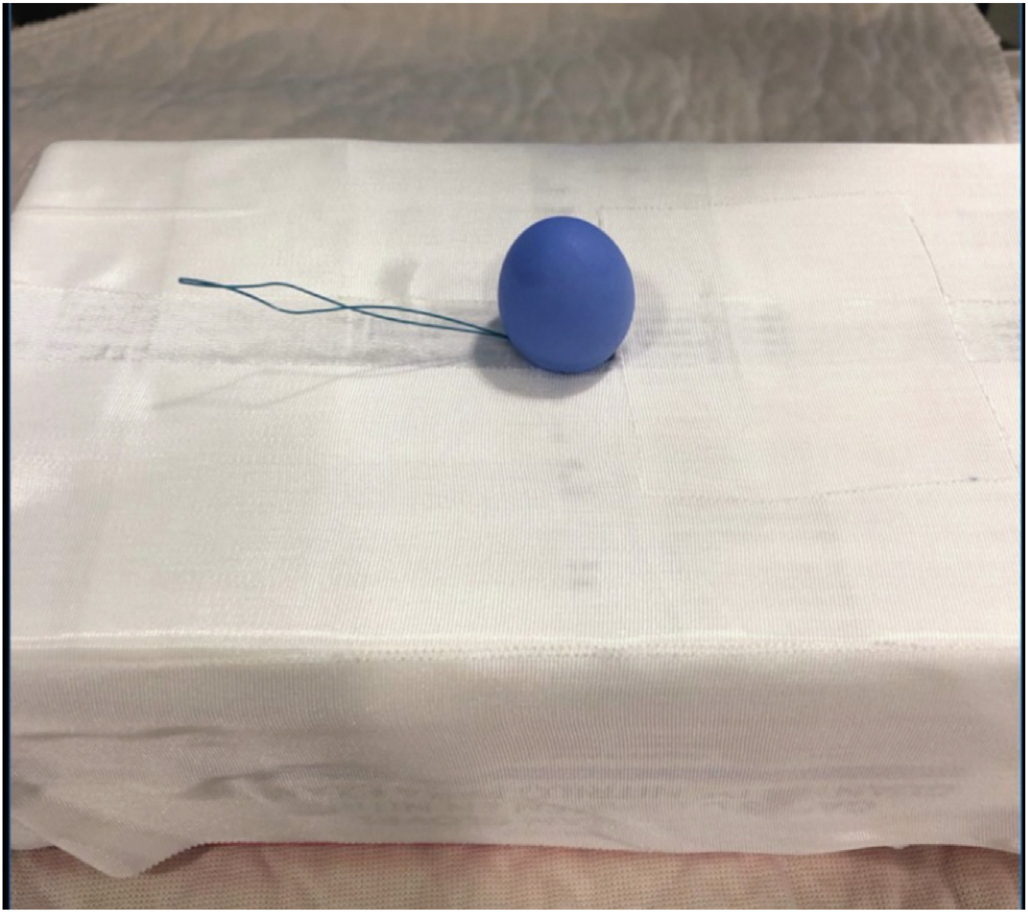
Model of loop-and-let-go technique. The device is placed over a simulated polyp, and the loop is closed snugly around the base. Once the device is in proper position, a cinch is deployed and the loop is detached and left in place.

**Figure 4:**
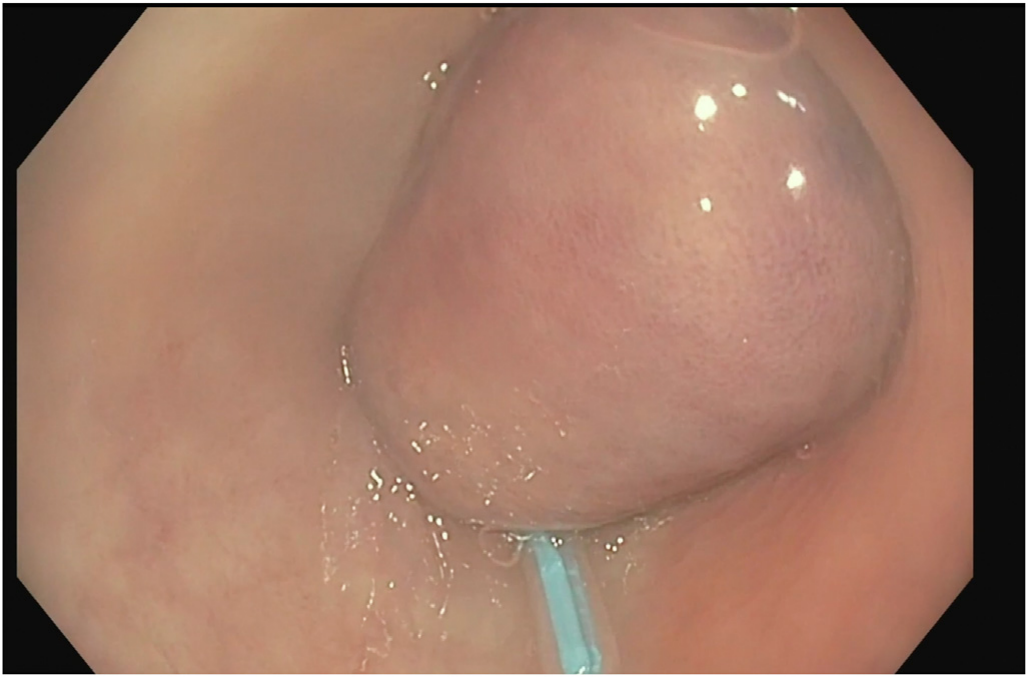
A detachable loop ligature device was successfully placed around the base of the lipoma.

Colonoscopy revealed a large polypoid mass, 50 mm in diameter, in the sigmoid colon (Fig. 1; Video 1, available online at www.videogie.org). Probing with biopsy forceps demonstrated a positive "pillow sign,” and bite-on-bite biopsies revealed underlying yellow tissue, suggestive of lipoma (Video 1). Radial echo endoscopy demonstrated a hyperechoic mass consistent with lipoma (Fig. 2; Video 1). Because of symptoms of intermittent obstruction and intussusception, the decision was made to proceed with removal. To minimize risk of perforation and adverse events, the decision was made to proceed with the loop-and-let-go technique. The stalk was injected with a total of 2 mL of epinephrine with blanching to reduce the likelihood of subsequent bleeding. A detachable loop ligature device (Fig. 3) was successfully placed around the base of the lipoma (Fig. 4; Video 1). The device was then cinched tightly and deployed. Once the device was placed, the ligature was left with a long redundant “tail.” This may be cut using a cutter to prevent entanglement if an emergent second flexible sigmoidoscopy is required after loop deployment. The lipoma was observed for 5 minutes and began displaying purple discoloration consistent with expected ischemia. The patient was recommended to return in 4 weeks for a repeat colonoscopy.

**Figure 5:**
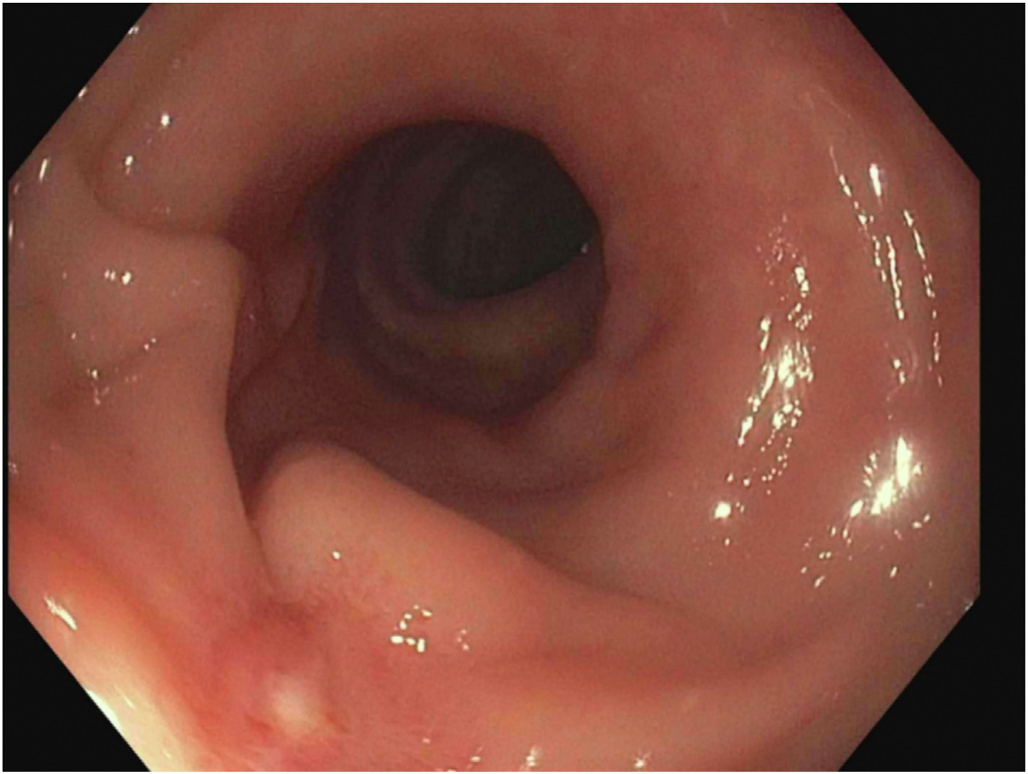
One small healing ulcer at the site of resection was seen with no stigmata of bleeding.

The patient was instructed to monitor bowel movements for passage of the lipoma in the stool. She did not report interval passage of the lipoma, abdominal pain, or bleeding. A follow-up endoscopy 4 weeks later revealed no endoscopic evidence of the previously seen lipoma. One small healing ulcer at the site of resection was seen with no stigmata of bleeding (Fig. 5). The patient’s abdominal symptoms of intermittent abdominal pain, bloating, and constipation subsequently resolved at both the follow-up endoscopy and at a clinic follow-up visit several months after the index procedure.

## Conclusion

Large colon lipomas can be challenging to resect endoscopically. EMR may carry the risk of perforation. This case demonstrates successful endoscopic removal of a large colonic lipoma using the loop-and-let-go technique with a detachable loop ligation. Strangulation of the base of the lipoma results in ischemia and eventual auto-amputation of the lesion, with low risk of perforation. A recent case series using this technique demonstrated greater than 90% success rate and no adverse events; however, all lesions included in the study were less than 2 cm. Our case suggests this technique can safely be applied to larger lesions, but diagnostic workup confirming the lipoma (such as EUS or pre-resection sampling) is recommended.

Although not performed here, a concurrent “unroofing technique” may be used following snare resection to remove residual lipomatous tissue. A recent systemic review found unroofing to be a safe approach, with low risk of perforation,^3^ although it may lead to incomplete removal of the tumor.^6^ A plausible approach would be to use a hybrid method with primary loop-and-let-go and subsequent “re-roofing” of residual tissue with a knife or snare after successful ligation.

## Disclosure

*The authors did not disclose any financial relationships.*

## Supplementary data

Video 1 We depict (1) identification of a large lipoma in the sigmoid colon; (2) radial echo endoscopy confirming diagnosis; (3) removal with the loop-and-let-go technique; (4) ex vivo demonstration of the technique; and (5) an interval follow-up demonstrating healing of the lesion.
